# Effectiveness of a Dual-Task Intervention Involving Exercise and Vocalized Cognitive Tasks

**DOI:** 10.3390/jcm13102962

**Published:** 2024-05-17

**Authors:** Masahiro Abo, Toyohiro Hamaguchi

**Affiliations:** 1 Department of Rehabilitation Medicine, Jikei University School of Medicine, Tokyo 105-8461, Japan; 2 Department of Rehabilitation, Graduate School of Health Science, Saitama Prefectural University, Saitama 343-8540, Japan; hamaguchi-toyohiro@spu.ac.jp

**Keywords:** cross-step training, dual task, MoCA-J, CS-30

## Abstract

**Background/Objectives**: Population aging is rapidly increasing, and the importance of preventive medicine has been stressed. Health checkups, diet, and exercise are of paramount importance. This study aimed to evaluate the effectiveness of a personalized dual-task intervention that combined exercise with cognitive tasks in improving physical and cognitive functions among independently living older individuals. **Methods**: Participants aged >65 years who were mostly independent in their activities of daily living were divided into two groups. The group receiving the 20 min robot-assisted session was compared with the group receiving traditional functional restoration training. This randomized trial assessed the impact of this intervention on the 30 s chair stand test score and Montreal Cognitive Assessment—Japanese version score of the participants. **Results**: Both scores significantly improved in the intervention group, indicating enhanced lower-limb function and cognitive capabilities. **Conclusions**: These findings suggest that integrating cognitive tasks with physical exercise can stand as an effective strategy to improve overall well-being in older people, offering valuable insights for designing comprehensive preventive health programs tailored to this demographic.

## 1. Introduction

Life expectancy is steadily increasing worldwide. The same holds true for Japan, where Japanese men and women had an average life expectancy of 81.05 years and 87.09 years, respectively, in 2022, making the Japanese the longest-living citizens globally [1]. Although an increase in life expectancy is a welcome development, it poses some medical challenges. The difference between the healthy life expectancy and average life expectancy in Japan is approximately 9 years for men and 12 years for women [2]. Currently, the Japanese government is implementing various measures to narrow the gap between the healthy life expectancy and average life expectancy. Disease prevention and management are indubitably important. In particular, regular effective exercise, which is crucial for disease prevention and management, is recommended to extend the healthy life expectancy [3]. Exercise interventions for patients with mild cognitive impairment (MCI), one of the issues affecting older people, do not seem to substantially improve the cognitive functions, except for the language function [4], but they have been shown to slow the decline in cognitive function [5,6], maintain and improve the patients’ activities of daily living, and improve the caregivers’ quality of life [7].

In Japan, the level of independence in daily living among older individuals at home is assessed using a six-point index based on disability severity. The government subsidizes the costs of assistance provided for household chores, daytime rehabilitation training, and home clinical care. A 2-week functional recovery training program is also offered to individuals who are deemed to require assistance, primarily because of osteoarticular disease, and who mostly exhibit independence in daily living. This training program aims to improve or maintain motor and cognitive functions and mainly involves gait training (e.g., stretching exercises), muscle training, aerobic training, and cross-step training.

This study specifically targeted the improvement of both physical and cognitive functions through a novel approach that integrates vocalized cognitive tasks with physical exercise. In recent years, interventions using dual tasks have been shown to maintain and improve functions in older people [8]. This study aimed to demonstrate that a combined approach could lead to synergistic improvements in motor function and cognitive resilience, especially in older people who are independent yet at risk of cognitive decline. Our primary hypothesis was that dual-task interventions, which integrate physical exercise with cognitive challenges, will produce greater improvements in both cognitive function and physical fitness in older adults when compared with single-task physical exercise alone. We hypothesized that this integrated approach would enhance attention, memory, and executive function alongside physical endurance and balance.

## 2. Materials and Methods

### 2.1. Study Design

This multicenter, prospective intervention study adopted a randomized trial design and enrolled outpatients from April 2023 to October 2023 at seven different hospitals in Japan. Patient data were collected from Izumi Memorial Hospital, Kawakita Sogo Hospital, Nishi-Hiroshima Rehabilitation Hospital, Sogo Tokyo Hospital, Kyoto Konoe Rehabilitation Hospital, Kyoto O’Hara Memorial Hospital, and Aomori Shin-Toshi Hospital and were subsequently analyzed.

### 2.2. Participants

The inclusion criteria were as follows: patients aged >65 years who were mostly independent in their activities of daily living. The participants were unaware of their group assignments, the study hypotheses, and the primary outcome measures.

At each institution, the patients were randomly assigned using a random number table using the Excel Rnd function (Microsoft, version 16) to either the robot-assisted therapy group (cross-step exercises in which the participants read aloud the questions presented on the screen, contemplated them, and then responded) or the conventional therapy group (cross-step exercises only). For blinding, the study design included the blinding of data analysts and the use of symbols to conceal group assignment from the researchers until the point of allocation. As for the intervention uniformity across hospitals, each hospital followed a standardized protocol for both robotic and conventional therapies to maintain consistency.

For a stratified analysis, the patients were divided into two groups (namely, mild and severe) according to baseline (pretreatment) severity, as assessed using the 30 s chair stand test (CS-30) score and Montreal Cognitive Assessment—Japanese version (MoCA-J) score. Differences between the intervention groups were analyzed using G*Power, with repeated-measures analysis of covariance (between factors) with the following values: effect size *f* = 0.30 [9], α = 0.05, 1-β = 0.80, and number of measurements = 3. Power analysis indicated that the required number of study patients was 88 (22 × 4 groups).

### 2.3. “Robot-Assisted” and Conventional Therapy Programs

The participants were patients who visited one of the seven hospitals. The group receiving the 20 min robot-assisted session was compared with the group receiving traditional functional restoration training at these hospitals. Traditional functional recovery training is a comprehensive, multidisciplinary team approach to functional recovery that provides rehabilitation and disability management services to individuals. Our team included physicians, physical therapists, occupational therapists, and speech therapists, and the functional rehabilitation program was customized with a disability management program tailored to the specific needs of the patients. Exercises and daily living guidance were provided for 40 min each to prevent patients from developing movement disorders and to enable them to safely perform various activities at work and in daily life. Assessments were conducted before, during, and after the start of the study. During the study period, each participant underwent a total of 8 training sessions twice a week, excluding evaluation days and Sundays.

**Figure 1:**
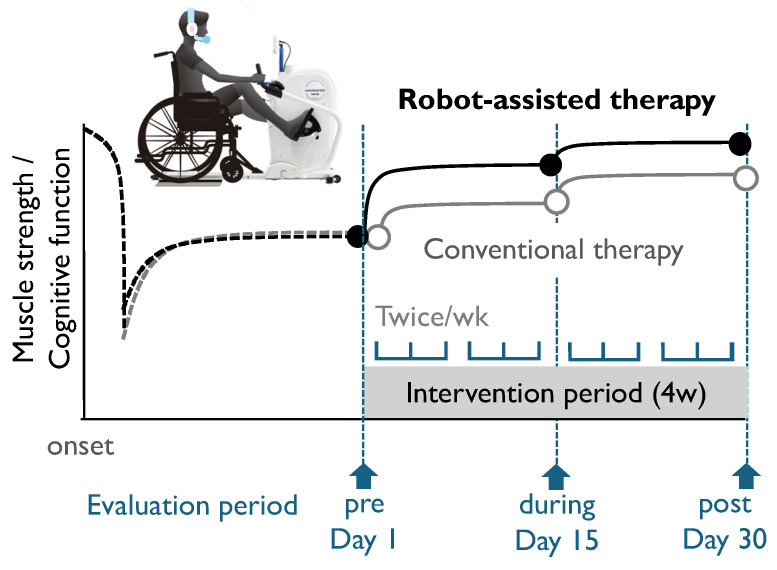
Conceptual diagram and protocol of this study designed to examine the differences in the effects of robot-assisted and conventional therapies on physical and cognitive functions in independently living older participants.

The equipment used for the 20 min robot-assisted session was a Cross Step WE-100 (OG Wellness, Okayama, Japan), which is designed to facilitate simultaneous movements of the upper and lower limbs, offering optimal aerobic exercise for older people. Physical training included upper-limb movements with the left and right arms gripping the handles, as well as simultaneous lower-limb movements with the left and right feet placed on the foot pedals (Figure 1). The alternating movements of the arms and lower extremities were coordinated with the movements of the entire body, thereby forcing the exercise of the major limbs and other body muscle groups. The pulse rate was recorded at the auricle throughout the 20 min exercise.

The exercise machine was connected to a personal computer that displayed questions on an LCD screen and provided voice instructions. Each participant wore a microphone with headphones and responded by following the voice instructions while viewing the LCD display.

The Cross Step device offers four exercise modes. In this study, the “multitasking mode”, which allows the user to set upper and lower heart rate limits and perform controlled exercises within that range, was selected. The upper pulse rate limit was set as 200 minus the age in years, the upper heart rate limit was set as the upper pulse rate minus 20, and the lower pulse rate limit was set as the lower pulse rate minus 40. The target heart rate for this study was set at 100–110 beats per minute.

Because the exercise duration was set at 20 min, the initial 30 s were set for warm-up, during which coordinated movements of the upper and lower limbs were performed. The countdown for the remaining 20 min commenced after the heart rate increased above the preset lower pulse rate limit. During this period, the LCD display flashed “warm up”, and instructions for responding to the questions were provided through the microphone and headphones. After the warm-up phase, the task mode began, and the task question was presented to the participants to be read aloud and answered. As the question was read, the time remaining to respond was displayed in the upper-left corner of the screen. Each participant was instructed to answer within this period. Failure to answer before the time limit expired was marked as an incorrect answer, and the system proceeded to the next question. This process continued throughout the 20 min exercise session, with participants alternating the movements of their upper and lower extremities while vocalizing their responses to the task displayed on the LCD screen. During the cool-down phase, the term “cool down” was flashed above the exercise time displayed on the screen.

The problems presented were generated by private educational organizations that support education in Japan. A total of 550 arithmetic, Japanese, and puzzle problems were provided by Gaudia (Gaudia Co., Kanagawa, Japan), and a total of 281 social study questions were supplied by Nichinokenkanto (Nichinokenkanto Co., Kanagawa, Japan). The difficulty level of these questions was set at the fifth-grade elementary school level. For instance, consider the following problem scenario: Japan comprises 47 prefectures. The shape of one of these prefectures was deliberately rotated, altering the typical map orientation. Subsequently, the older participants in training were instructed to identify which prefecture was depicted on the map. In another problem, the middle kanji character was removed from a 3 × 3 grid, and the older participants were prompted to determine which kanji character could be inserted to form a two-character idiom in conjunction with the surrounding kanji characters. Each question was meticulously crafted by three rehabilitation specialists to require not only memorization but also engagement of at least one of the following cognitive functions: executive function, attention, orientation, and visuospatial cognition.

The system was configured to allow the participants to answer a minimum of six questions within a 30 s timeframe for each question on average and a maximum of twelve questions if they were answered on average every 15 s during the exercise. The questions were randomly selected from those registered in the designated fields.

### 2.4. Variables and Timing

The participants were divided into intervention and control groups to verify the effectiveness of the robot-assisted therapy. Progress was investigated at three time points (Figure 1). Sarcopenia, defined as low muscle strength, was evaluated using the CS-30, which is particularly useful for assessing muscle strength as the main outcome [10]. The chair stand test has been confirmed to be useful for evaluating muscle strength and physical performance [11,12]. The CS-30 score correlated with sarcopenia (odds ratio: 0.88; 95% confidence interval [95% CI]: 0.82–0.93). The optimal number of stands in the CS-30 that predicted sarcopenia was 15 for females (sensitivity, 76.4%; specificity, 76.8%) and 17 for males (sensitivity, 75.0%; specificity, 71.7%) [10]. The patients were evaluated on admission (pre), during the intervention (during), and after the intervention (post) using the CS-30.

As for the secondary outcome, the MoCA-J score was utilized to determine the presence or absence of MCI, using a MoCA-J score of <26 as the threshold [13,14]. For a stratified analysis, patients in the intervention and control groups were divided into a severe group (sarcopenia, CS-30 score < 17 for men and <15 for women) and a mild/moderate cognitive impairment group (MoCA-J score < 26). All patients were diagnosed by attending physicians at each hospital. A licensed physical therapist or occupational therapist performed all screening and testing procedures.

### 2.5. Statistical Analysis

The CS-30 and MoCA-J scores were used as objective variables in the repeated-measures analysis of variance (ANOVA) and post hoc *t*-tests for pairwise comparisons. Sex and age were used as covariates in the ANOVA. Due to the presence of a quadratic variable of different hospitals participating in the study, a linear mixed model analysis with a random intercept of hospitals was conducted. This analysis aimed to test whether the variation between hospitals affected the results and to ensure the robustness and generalizability of the findings. Analyses were performed using Jeffreys’s Amazing Statistics Program (JASP) software version 0.18.1 (https://jasp-stats.org). Post hoc comparisons were conducted using Tukey’s post hoc correction. All objective variables were tested for data equality using Levene’s test and Mauchly’s sphericity test. A *p*-value of <0.05 denoted the presence of a statistically significant difference.

### 2.6. Ethical Considerations

Informed consent from patients was not required for the analysis because it used anonymous clinical data obtained after each patient gave written consent for treatment. The opt-out method was employed to obtain consent using a poster and website approved by the Institutional Review Board of Tokyo Jikei University Hospital (approval number: #32-338(10423); date of approval: 9 November 2021). In terms of ethical considerations, the confidentiality and privacy of patient data were upheld through several measures. All data were anonymized at the point of collection, and access to the data was restricted to the research team only. Data were stored in encrypted forms, with regular audits to ensure compliance with data protection regulations.

## 3. Results

### 3.1. Participants

**Figure 2:**
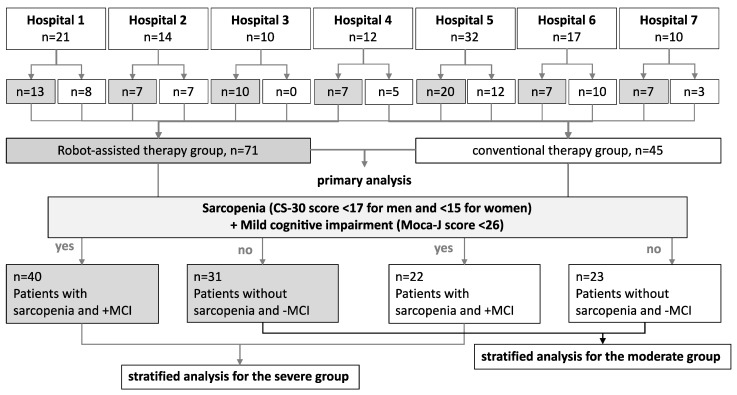
Flow chart of the processing protocol applied in this study for the inclusion and categorization of the participants. The included study participants from seven hospitals (*n* = 116) underwent robot-assisted or conventional therapy. The same patients were also divided into two groups according to the presence or absence of sarcopenia and MCI.

During the study period, rehabilitation was prescribed to 116 patients with a history of various conditions (e.g., cerebrovascular diseases, vertebral fractures, lower-limb fractures, angina pectoris, hypertension, and hyperlipidemia) at all seven medical institutions. At each hospital, the patients were randomly assigned to either the robot-assisted or conventional therapy group. The number of patients varied among the participating hospitals and ranged from 10 to 32 per institution. All patients were included in this study (Table 1), and there were no dropouts. Data from all 116 patients were analyzed (Figure 2).

### 3.2. Descriptive Data

Table 1 summarizes the clinical characteristics of the patients. The numbers of patients randomly allocated to the robot-assisted and conventional therapy groups were 71 (61%) and 45 (39%), respectively. In addition to comparing the intervention groups, the robot-assisted and control groups were further divided into severe and moderate groups to perform a stratified analysis based on severity, as assessed using the presence or absence of sarcopenia and cognitive dysfunction (Figure 2).

### 3.3. Outcome Data

The CS-30 score (median, first to third quarters) before the intervention was 14 (range, 10–17) in the robot-assisted group and also 14 (range, 11–17) in the conventional therapy group. The total MoCA-J scores before the intervention were 22 (range, 20–24) in the robot-assisted group and 23 (range, 21–26) in the conventional therapy group. The Barthel Index score was very high in almost all patients, irrespective of background, intervention group, and severity (Table 1), indicating that they were independent in their daily self-care. The power grip, CS-30, MoCA-J, and Barthel Index scores did not differ between the robot-assisted and control groups; however, significant differences in these parameters were noted between the severe and moderate groups at baseline (Table 1). We conducted a linear mixed model analysis with random intercepts for hospitals to assess whether variations between the hospitals where this study was conducted influenced the results. We included hospital differences as a factor to examine their main effect (all *p* < 0.01, *F* [6, 103] = 7.76–9.24). In a linear mixed model analysis, there was no interaction of hospital x intervention period x intervention/non-intervention group (all *p* = 0.24–0.28, *F* [10, 206] = 1.22–1.29, AIC = 1842–1955).

### 3.4. Effects of the Interventions on the Lower-Limb Function

**Figure 3:**
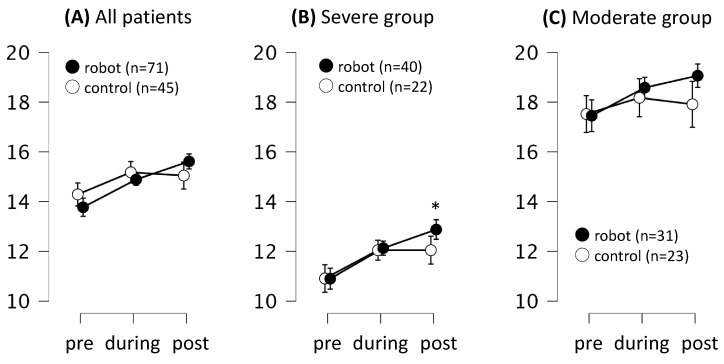
Effects of robot-assisted and conventional therapies on the CS-30 score. (**A**) Comparison of changes in the total score of all patients. (**B**) Stratified analysis of patients with sarcopenia (CS-30 score < 17 for men and < 15 for women) and MCI (MoCA-J score < 26). (**C**) Stratified analysis of patients without sarcopenia (CS-30 score ≥ 17 for men and ≥15 for women) and without MCI (MoCA-J score ≥ 26). The data show lower-limb function with means (circles) and standard deviations (error bars). *n* = 116, repeated-measures ANOVA, with sex and age as covariates. * vs. pre–post in the robot-assisted group. *p*_Tukey_ < 0.05 for post hoc comparisons.

A further analysis compared the effects of each intervention on the CS-30 score. The two interventions showed no significant differences in the changes in the lower-limb function, as assessed using the CS-30 score (time course × group interaction; *F* = 0.76, *p* = 0.46, η^2^ = 0.00, by Greenhouse–Geisser sphericity correction). The CS-30 score of the robot-assisted group increased by approximately 1.7 times post-intervention, and the improvement over time was significant relative to the pre-intervention score (mean difference = 1.7, 95% CI: 0.3–3.2, *t* = 3.57, *p*_Tukey_ = 0.01, Cohen’s *d* = 0.31). Similar results were obtained for the severe group (mean difference = 2.0, 95% CI: 0.4–3.6, *t* = 3.75, *p*_Tukey_ < 0.01, Cohen’s *d* = 0.56) but not for the moderate group (mean difference = 1.1, 95% CI: 3.8–5.9, *t* = 0.66, *p*_Tukey_ = 0.69, Cohen’s *d* = 0.23). No significant changes in the lower-limb function were observed in the conventional therapy group (*p* > 0.05; Figure 3 and Table 2).

### 3.5. Effects of the Interventions on the Cognitive Function

**Figure 4:**
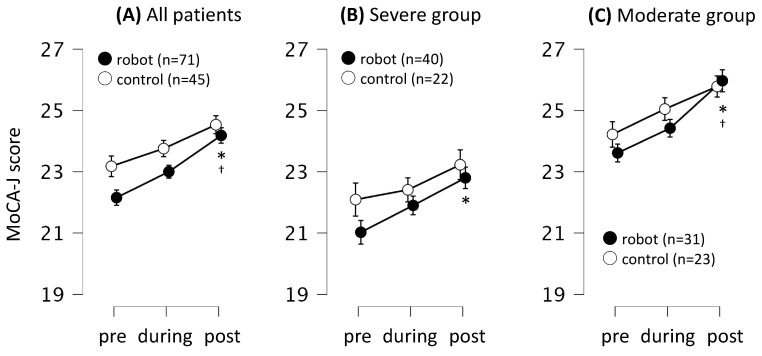
Effects of robot-assisted and conventional therapies on the MoCA-J score. (**A**) Comparison of changes in the total score. (**B**) Stratified analysis of patients with sarcopenia (CS-30 score < 17 for men and < 15 for women) and MCI (MoCA-J score < 26). (**C**) Stratified analysis of patients without sarcopenia (CS-30 score ≥ 17 for men and ≥15 for women) and without mild dementia (MoCA-J score ≥ 26). The data show cognitive function with mean values (circle dots) and standard deviations (error bars). *n* = 116, repeated-measures ANOVA, with sex and age as covariates. *n* = 116, * vs. pre–post. ^†^ vs. during–post in the robot-assisted group. *p*_Tukey_ < 0.05 for post hoc comparisons.

The effects of each intervention on cognitive function were also analyzed using the MoCA-J score. The trend for the changes in cognitive function was similar between the robot-assisted and conventional therapy groups (time course × group interaction; *F* = 0.71, *p* = 0.49, η^2^ < 0.01; Figure 4). In the robot-assisted group, the MoCA-J score was higher after the intervention than pre-intervention (mean difference = 2.0, 95% CI: 1.0–3.0, *t* = 5.74, *p*_Tukey_ < 0.01, Cohen’s *d* = 0.56). Furthermore, the MoCA-J score after the intervention was higher than that during the intervention (mean difference = 1.2, 95% CI: 0.1–2.2, *t* = 3.38, *p*_Tukey_ = 0.01, Cohen’s *d* = 0.33). The intervention increased the MoCA-J score in both the severe group (mean difference = 2.6, 95% CI: 0.1–3.0, *t* = 3.23, *p*_Tukey_ = 0.02, Cohen’s *d* = 0.54) and moderate group (mean difference = 2.6, 95% CI: 1.2–3.9, *t* = 5.73, *p*_Tukey_ < 0.01, Cohen’s *d* = 0.70) compared to the respective baseline values. Furthermore, the MoCA-J score of the moderate group after the intervention was higher than that during the intervention (mean difference = 1.6, 95% CI: 0.2–2.9, *t* = 3.48, *p*_Tukey_ = 0.04, Cohen’s *d* = 0.43). However, the changes in cognitive function observed in the conventional group were not statistically significant (*p* > 0.05) (Figure 4 and Table 3).

## 4. Discussion

This randomized trial revealed that our individualized training program tailored for independently living older adults resulted in improvements in the CS-30 and MoCA-J scores only in the dual-task intervention group. The CS-30, which is often used as a screening tool for sarcopenia in older Japanese individuals and correlates with lower-limb extensor muscle strength [10] showed a greater improvement in the intervention group with severe conditions (sarcopenia and MCI). The improvement observed in CS-30 scores in the robot-assisted group suggested improved lower-extremity function. The minimal clinically important difference in CS-30 scores was previously reported to be 2 for lung diseases [15] and orthopedic diseases [16]. The changes observed in CS-30 scores among the older, frail patients in this study suggested a clinically significant recovery. The MoCA-J score improved not only in the intervention group with severe conditions but also in the group with mild conditions (without sarcopenia and MCI). The minimal clinically important difference in MoCA was approximately 1.0–2.2, indicating an improvement in the MoCA score from the second week of the intervention [17]. While older Japanese individuals are known to have a slim physique, our findings suggest that combining cognitive training with conventional cross-step training is potentially highly effective in improving balance, posture, gait, and cognitive functions in older men and women. However, the frequency of exercise and the difficulty level of the cognitive task are very important [18,19]. Despite the effectiveness of the present task, it is important to optimize the frequency of exercise sessions and cognitive tasks to improve both cognitive and physical functions, while also exploring long-term effects.

Dual-task paradigms, which are primarily investigated in psychology, often focus on the assessment of changes following the completion of two simultaneous tasks. Previous studies reported the favorable outcomes of training on balance and gait across various populations, including older individuals [20,21,22] and patients with stroke [23]. Intervention studies also showed improvements in locomotion amplitude and velocity during walking after dual-task training in older individuals [20]. Moreover, independently living older individuals exhibited improvements in balance and gait performance with dual-task engagement. Notably, a previous study of independently living older participants who completed 21 sessions (40 min/session) of dual-task training over a 7-week period reported significant improvements in gait speed, lower-limb muscle strength, and reaction time in cognitive tasks (specifically, attention dispersion) [21]. Another study reported that older participants exhibited an improved walking ability after completing 45 min dual-task training sessions thrice weekly for 8 weeks [22]. However, the improvements observed after another dual-task intervention that incorporated verbal tasks in community-dwelling patients with stroke varied, with the Stroop dual-task intervention showing the most significant enhancement. Inconsistent results were also reported for the improvement in walking speed with time and speech tasks, which were not part of the training. The latter findings suggest a potential limitation in the translation of training effects to dual cognitive–motor tasks [23].

The improvements in lower limb and cognitive functions noted in the dual-task group compared with the single-task group are clinically significant for several reasons. First, enhancing lower-limb strength and coordination directly correlates with a reduced risk of a fall, which is a major concern for older adults [24]. Falls are a leading cause of injury and morbidity in this population, and improving motor function can significantly decrease the incidence of falls, thereby extending the healthy life years and reducing healthcare costs. Secondly, the cognitive improvements observed contribute to better executive functioning and memory processes, which are crucial for maintaining independence in daily activities [25]. As cognitive decline is one of the primary reasons why older adults lose their independence, interventions that can bolster cognitive health are extremely valuable [26]. Enhanced cognitive function helps older adults manage daily decisions, remember important medications and appointments, and maintain social interactions, which are vital for mental health and quality of life.

According to the task integration hypothesis, participants can develop coordination skills by practicing two tasks simultaneously rather than a single task. Efficient integration and coordination between the two tasks acquired during dual-task training are crucial for improving dual-task performance [27]. This implies that the activities assigned to participants in the dual-task training group (task + cognitive task) are significantly more challenging than those assigned to participants in the single-task training group (task only). The task integration hypothesis posits that engaging in activities that require simultaneous cognitive and physical exertion forces the brain to optimize its resource allocation [28]. This integration leads to improvements in both cognitive and motor functions as the brain learns to handle multiple demands more efficiently. This hypothesis is supported by recent neuroscientific research that demonstrates increased neural efficiency and enhanced connectivity in areas of the brain involved in multitasking. In a study that combined a response inhibition task with walking, we found that as behavior deteriorates with aging and improves during dual-task walking [29], EEG evidence of walking-related amplitude modulation in the frontocentral and left prefrontal regions was observed. These neural signatures of behavioral improvement may reflect a more flexible recalibration of neural processes related to the cognitive components of inhibition as task demands increase.

The simultaneous processing and execution of multiple tasks become increasingly challenging with age, particularly in individuals with cognitive decline or impairment. In addition to the processing capacity required for each task, the effective allocation and division of attention are crucial for the proper handling of multiple tasks. Balance control is commonly impaired under dual-task conditions in the older population. Given that compromised balance during dual-task situations can predict adverse outcomes such as falls [30,31], in addition to cognitive and physical decline, interventions aimed at enhancing balance during dual-task scenarios [32,33] have been recognized as important medical necessities in aging societies [34,35].

In this study, cross-step exercise training was performed. While cross-step exercise, aerobics, and treadmill walking are commonly advised for lower-limb training in the older population in Japan, we opted for the cross-step exercise to mitigate the risk of falls during dual-task performance. The cross-step equipment features a load setting that is adjustable according to the heart rate, ensuring that each participant receives a comparable training intensity. This approach aims to maintain consistent motor task levels, even after dual-task training sessions. A previous study suggested that the conduct of dual-task protocols, such as when patients with stroke are engaged simultaneously in voice and walking tasks, is often marred by cognitive–motor interference, with a resultant increase in execution time and fewer steps in any motor task owing to slower and reduced joint movements [36]. However, considering that our study entailed only 20 min of training sessions twice a week, we believe that the cognitive load was relatively low. Therefore, as a continuation of the method by which our study evaluated the aspects of gait, further investigations of gait and related parameters remain a potential area for future research.

Regarding the potential influence of hospital differences on the effectiveness of robotic rehabilitation interventions, our linear mixed model analysis did not reveal a statistically significant interaction effect between hospital, intervention period, and intervention/non-intervention group. This finding suggests that, while variations in patient outcomes can be observed across different hospitals, these variations do not significantly affect the overall effectiveness of the interventions administered. The lack of a significant interaction indicates that the differences in physical and cognitive function improvements can be attributed primarily to the interventions themselves rather than the hospital settings or patient demographics specific to each hospital. Therefore, the results support the robustness of the robotic intervention across different hospital environments, reinforcing its potential for broader implementation.

In Japan, compulsory education spans nine years from elementary to junior high school, providing individuals with foundational knowledge aligned with educational guidelines. The cognitive tasks used in this study were developed at a Japanese educational institution. During the initial sessions of the actual study, when the participants read and answered the questions aloud while performing the cross-step exercises, it was observed that the questions for which they managed to provide correct answers were not at the junior high school level but, rather, at the fifth-grade elementary school level. Hence, the difficulty level of the questions was set to the fifth-grade elementary school level. These cognitive tasks encompassed inhibitory control tasks (e.g., alternating letters of the alphabet, auditory selection responses) and working memory tasks (e.g., sequential subtraction, fluent verbal utterances, reverse spelling) [37]. Even tasks that could not be solved required considerable effort from the older participants, particularly those demanding inhibitory control. Consequently, it can be inferred that brain hemodynamics during exercise are likely influenced by dual-task demands.

We recently reported that aphasia in patients who are independent in their daily lives can be improved through repetitive high-frequency transcranial magnetic stimulation and speech and hearing training targeted at areas of language activation identified during a recitation task using functional magnetic resonance imaging [38]. It is noteworthy that patients with aphasia and higher brain dysfunction benefit from responding aloud to tasks rather than simply contemplating them internally. This approach offers the advantage of identifying sites of language activation. Therefore, for older patients who may experience difficulties with higher brain functions, engaging in tasks involving reading, comprehending, and responding aloud during exercise is highly meaningful. In addition, even tasks deemed unsolvable may affect cerebral hemodynamics during exercise, particularly in dual-task scenarios, given that older individuals expend considerable efforts to complete tasks, especially those requiring inhibitory control.

This study had several limitations. For instance, training was conducted twice a week and comprised 20 min sessions for ease of participation, though most reports on dual-task training programs are based on a long-term follow-up of approximately six months, with training sessions lasting for 45–60 min at least three times a week. This highlights that both the frequency of training sessions and the duration of training should be considered when comparing the effectiveness of different training programs reported in the literature. It is also possible that the older participants in our study regularly visited the hospital for appointments related to other illnesses on the days that they attended training, suggesting that their focus might have been split between their participation in this program and tasks other than those in the training program. Additionally, we used the CS-30 only to measure the performance under dual-task conditions. In future studies, if the number of training sessions is increased, the assessment of balance during walking should be correlated with improvements in fall prevention using more comprehensive measures of physical performance, such as gait speed and the inclination angle between the center of gravity and the center of pressure. This should provide a better understanding of the effectiveness of both dual- and single-task training, and whether dual-task training programs are effective in improving single-task performance should be clarified. While we accounted for potential confounders such as age, sex, and severity of physical and cognitive functional impairment in our analysis, it is important to note that we did not perform adjustments for individual baseline values of the primary outcomes. This decision was based on our initial study design, which emphasized adjustments for broad demographic factors to maintain a manageable complexity in our statistical model. However, we recognize that this limited our ability to fully control for all initial disparities between the experimental and control groups, potentially influencing the intervention effects observed. Our small effect sizes also suggest that larger sample sizes are needed for future studies to adequately note the effects, or they might prompt a reevaluation of the research methods or measures used. Finally, a linear mixed model analysis of the differences between hospitals in the results of this study suggested that there were differences in the physical and cognitive functions of patients between hospitals. It was inferred that the hospitals where this study was conducted exhibited variations in patient illness severity, likely due to their distinct roles in community healthcare within Japan. After considering hospital differences, we observed the effects of robotic therapy on physical and cognitive functions, though further validation is required at other facilities catering to different patient demographics.

## 5. Conclusions

Our study showed that individualized dual-task training combining traditional interventions with a range of cognitive tasks was feasible for community-dwelling older individuals who were somewhat independent in their daily lives. Moreover, the participants successfully complied with instructions regarding attentional focus, directing their attention effectively towards the specified tasks and leading to measurable improvements in cognitive function. The results obtained from taking this approach may be broadly applicable to older adults. In view of the Japanese elderly care or medical health system, the limited scope of interventions in terms of intervention time and frequency should be considered. Future research should explore the impact of varying these parameters to optimize the effectiveness of dual-task interventions stratified by the severity of dementia and physical disability. Additionally, long-term follow-up studies are warranted to assess the sustainability of the observed effects and evaluate the potential of these interventions in preventing functional independence decline in older individuals. These future investigations may significantly help in refining dual-task intervention programs tailored to the needs of the aging population, ultimately improving their health and life expectancy.

## Figures and Tables

**Table 1:** Descriptive statistics of all patients at baseline (pre-intervention).

Characteristics	All Patients (*n* = 116)		Dual-Task Group (*n* = 71)		Control (*n* = 45)		Statistics for Severe vs. Moderate
	Dual-Task Group	Control	Severe	Moderate	Severe	Moderate	
Number of patients	70	45	40	31	22	23	χ^2^ = 116, *p* = 0.456
Age (years)	76 (70–80)	79 (73–86)	78 (72–81)	72 (67–80)	80 (73–87)	78 (74–83)	*F* = 5.03, *p* = 0.03, η^2^ = 0.04
Sex (females:males)	40:31	24:21	22:18	18:13	10:12	14:9	χ^2^ = 0.10, *p* = 0.751
Grip power							
Right side, kg	23.7 (19.4–28.0)	22.3 (15.5–28.5)	22.3 (18.4–24.1)	25.5 (20.1–31.1)	21.8 (14.8–29.6)	22.7 (17.8–27.1)	*F* = 2.09, *p* = 0.15, η^2^ = 0.04
Left side, kg	19.8 (15.3–24.5)	20.0 (14.5–25.5)	19.1 (14.5–21.0)	20.7(17.1–25.1) *	17.6 (13.3–18.9)	22.3 (16.8–27.9) *	*F* = 6.12, *p* = 0.02, η^2^ = 0.05
30 s chair stand test	14 (10–17)	14 (11–17)	11 (9–13)	17 (13–21) *	11 (9–13)	18 (14–19) *	*F* = 78.52, *p* < 0.001, η^2^ = 0.40
Montreal Cognitive Assessment	22 (20–24)	23 (21–26)	21 (19–23)	24 (21–27)	22 (21–23)	24 (21–27)	*F* = 15.23, *p* < 0.001, η^2^ = 0.12
Barthel Index	99 (100–100)	99 (100–100)	100 (100–100)	99 (100–100)	99 (96–100)	99 (100–100)	*F* = 0.03, *p* = 0.864, η^2^ = 0.00

Data are presented as medians and 25th–75th-percentile values. The chi-squared test was used to compare the number of patients in each group. Group differences in severity were tested using analysis of variance (grip power, 30 s chair stand test, Montreal Cognitive Assessment, and Barthel Index). The left and right sides represent the affected sides. * *p* < 0.05 for Tukey’s post hoc test comparing the severe and moderate groups. In statistics for severe vs. moderate, *p*-values present the results of comparisons between the dual-task and control groups.

**Table 2:** Description of physical and cognitive status in the intervention groups.

Time Course	Robot (*n* = 71)	Control (*n* = 45)	Repeated-Measures ANOVA (Time Course × Group)
CS-30			
Pre	13.8 ± 5.0	14.3 ± 5.1	†*F* = 0.58, *p* = 0.46, η^2^ = 0.01
During	14.9 ± 5.5	15.2 ± 6.3	Assumption checks
Post	15.6 ± 5.8 *	15.0 ± 6.2	Mauchly’s *W* = 0.91, *p* = 0.01
MoCA-J			
Pre	22.2 ± 3.4	23.2 ± 3.3	*F* = 0.71, *p* = 0.49, η^2^ = 0.00
During	23.0 ± 3.7	23.8 ± 4.1	Assumption checks
Post	24.2 ± 3.6 *†	24.5 ± 3.7	Mauchly’s *W* = 0.96, *p* = 0.09

Data are presented as mean ± standard deviation. All severity data for each item were estimated statistically using repeated-measures ANOVA, with sex and age as factors. † Greenhouse–Geisser sphericity correction was used because Mauchly’s test was violated (*p* < 0.05). * vs. pre–post in the robot-assisted group. † vs. during–post. *p*_Tukey_ < 0.05 for intragroup comparisons.

**Table 3:** Description of physical and cognitive status in the patient groups by severity.

Time Course	Severe		Repeated-Measures ANOVA Time Course × Group	Moderate		Repeated-Measures ANOVA Time Course × Group
	Robot (*n* = 40)	Control (*n* = 22)		Robot (*n* = 31)	Control (*n* = 23)	
CS-30						
Pre	10.9 ± 2.6	10.9 ± 2.5	†*F* = 0.58, *p* = 0.54, η^2^ = 0.00. Assumption checks; Mauchly’s *W* = 0.84, *p* = 0.01	17.5 ± 5.1	17.5 ± 4.9	*F* = 0.26, *p* = 0.77, η^2^ = 0.00. Assumption checks; Mauchly’s *W* = 0.95, *p* = 0.29
During	12.1 ± 3.8	12.0 ± 3.8		18.6 ± 5.4	18.2 ± 6.7	
Post	12.9 ± 4.5 *	12.0 ± 3.9		19.1 ± 5.4	17.9 ± 6.6	
MoCA-J						
Pre	21.0 ± 2.4	22.1 ± 2.4	*F* = 0.02, *p* = 0.98, η^2^ = 0.00. Assumption checks; Mauchly’s *W* = 0.93, *p* = 0.13	23.6 ± 3.9	24.2 ± 3.7	*F* = 2.3, *p* = 0.11, η^2^ = 0.01. Assumption checks; Mauchly’s *W* = 0.95, *p* = 0.84
During	21.9 ± 3.0	22.4 ± 3.7		24.4 ± 4.1	25.0 ± 4.1	
Post	22.8 ± 3.4 *	23.2 ± 3.7		26.0 ± 3.0 *†	25.8 ± 3.4	

Data are presented as mean ± standard deviation. All severity data for each item were estimated statistically using repeated-measures ANOVA, with sex and age as factors. † Greenhouse–Geisser sphericity correction was used because Mauchly’s test was violated (*p* < 0.05). * vs. pre–post in the robot-assisted group. † vs. during–post. *p*_Tukey_ < 0.05 for intragroup comparisons.

## Data Availability

The data that support the findings of this study are available from the corresponding author, M.A., upon reasonable request.

## References

[1] Director-General for Statistics, Information System Management and Industrial Relations Ministry of Health, Labour and Welfare Government of Japan (2022). Abridged Life Tables for Japan 2022.

[2] Ministry of Health, Labour and Welfare (2021). Vital Statistics.

[3] Myers J., Prakash M., Froelicher V., Do D., Partington S., Atwood J.E. (2002). Exercise capacity and mortality among men referred for exercise testing. N. Engl. J. Med..

[4] Gates N., Fiatarone Singh M.A., Sachdev P.S., Valenzuela M. (2013). The effect of exercise training on cognitive function in older adults with mild cognitive impairment: A meta-analysis of randomized controlled trials. Am. J. Geriatr. Psychiatry.

[5] Venturelli M., Scarsini R., Schena F. (2011). Six-month walking program changes cognitive and ADL performance in patients with Alzheimer. Am. J. Alzheimers Dis. Other Demen..

[6] Yuan F., Klavon E., Liu Z., Lopez R.P., Zhao X. (2021). A Systematic Review of Robotic Rehabilitation for Cognitive Training. Front. Robot AI.

[7] Barnes D.E., Mehling W., Wu E., Beristianos M., Yaffe K., Skultety K., Chesney M.A. (2015). Preventing loss of independence through exercise (PLIE): A pilot clinical trial in older adults with dementia. PLoS ONE.

[8] Jardim N.Y.V., Bento-Torres N.V.O., Costa V.O., Carvalho J.P.R., Pontes H.T.S., Tomas A.M., Sosthenes M.C.K., Erickson K.I., Bento-Torres J., Diniz C.W.P. (2021). Dual-Task Exercise to Improve Cognition and Functional Capacity of Healthy Older Adults. Front. Aging Neurosci..

[9] Cohen J. (1988). Statistical Power Analysis for the Behavioral Sciences.

[10] Sawada S., Ozaki H., Natsume T., Deng P., Yoshihara T., Nakagata T., Osawa T., Ishihara Y., Kitada T., Kimura K. (2021). The 30-s chair stand test can be a useful tool for screening sarcopenia in elderly Japanese participants. BMC Musculoskelet. Disord..

[11] Chen L.K., Woo J., Assantachai P., Auyeung T.W., Chou M.Y., Iijima K., Jang H.C., Kang L., Kim M., Kim S. (2020). Asian Working Group for Sarcopenia: 2019 Consensus Update on Sarcopenia Diagnosis and Treatment. J. Am. Med. Dir. Assoc..

[12] Cruz-Jentoft A.J., Bahat G., Bauer J., Boirie Y., Bruyere O., Cederholm T., Cooper C., Landi F., Rolland Y., Sayer A.A. (2019). Sarcopenia: Revised European consensus on definition and diagnosis. Age Ageing.

[13] Fujiwara Y., Suzuki H., Yasunaga M., Sugiyama M., Ijuin M., Sakuma N., Inagaki H., Iwasa H., Ura C., Yatomi N. (2010). Brief screening tool for mild cognitive impairment in older Japanese: Validation of the Japanese version of the Montreal Cognitive Assessment. Geriatr. Gerontol. Int..

[14] Nasreddine Z.S., Phillips N.A., Bedirian V., Charbonneau S., Whitehead V., Collin I., Cummings J.L., Chertkow H. (2005). The Montreal Cognitive Assessment, MoCA: A brief screening tool for mild cognitive impairment. J. Am. Geriatr. Soc..

[15] Zanini A., Crisafulli E., D’Andria M., Gregorini C., Cherubino F., Zampogna E., Azzola A., Spanevello A., Schiavone N., Chetta A. (2019). Minimum Clinically Important Difference in 30-s Sit-to-Stand Test After Pulmonary Rehabilitation in Subjects with COPD. Respir. Care.

[16] Wright A.A., Cook C.E., Baxter G.D., Dockerty J.D., Abbott J.H. (2011). A comparison of 3 methodological approaches to defining major clinically important improvement of 4 performance measures in patients with hip osteoarthritis. J. Orthop Sports Phys. Ther..

[17] Wu C.Y., Hung S.J., Lin K.C., Chen K.H., Chen P., Tsay P.K. (2019). Responsiveness, Minimal Clinically Important Difference, and Validity of the MoCA in Stroke Rehabilitation. Occup. Ther. Int..

[18] Forte R., Trentin C., Tocci N., Lucia S., Aydin M., Di Russo F. (2023). Motor-cognitive exercise with variability of practice and feedback improves functional ability and cognition in older individuals. Aging Clin. Exp. Res..

[19] Chen I.C., Chuang I.C., Chang K.C., Chang C.H., Wu C.Y. (2023). Dual task measures in older adults with and without cognitive impairment: Response to simultaneous cognitive-exercise training and minimal clinically important difference estimates. BMC Geriatr..

[20] van het Reve E., de Bruin E.D. (2014). Strength-balance supplemented with computerized cognitive training to improve dual task gait and divided attention in older adults: A multicenter randomized-controlled trial. BMC Geriatr..

[21] Adcock M., Sonder F., Schattin A., Gennaro F., de Bruin E.D. (2020). A usability study of a multicomponent video game-based training for older adults. Eur. Rev. Aging Phys. Act..

[22] Azadian E., Torbati H.R., Kakhki A.R., Farahpour N. (2016). The effect of dual task and executive training on pattern of gait in older adults with balance impairment: A Randomized controlled trial. Arch. Gerontol. Geriatr..

[23] Bayouk J.F., Boucher J.P., Leroux A. (2006). Balance training following stroke: Effects of task-oriented exercises with and without altered sensory input. Int. J. Rehabil. Res..

[24] Begde A., Alqurafi A., Pain M.T.G., Blenkinsop G., Wilcockson T.D.W., Hogervorst E. (2023). The Effectiveness of Home-Based Exergames Training on Cognition and Balance in Older Adults: A Comparative Quasi-Randomized Study of Two Exergame Interventions. Innov. Aging.

[25] Hong X.L., Cheng L.J., Feng R.C., Goh J., Gyanwali B., Itoh S., Tam W.S.W., Wu X.V. (2024). Effect of physio-cognitive dual-task training on cognition in pre-ageing and older adults with neurocognitive disorders: A meta-analysis and meta-regression of randomized controlled trial. Arch. Gerontol. Geriatr..

[26] Li F., Harmer P., Eckstrom E., Fitzgerald K., Winters-Stone K. (2023). Clinical Effectiveness of Cognitively Enhanced Tai Ji Quan Training on Global Cognition and Dual-Task Performance During Walking in Older Adults With Mild Cognitive Impairment or Self-Reported Memory Concerns: A Randomized Controlled Trial. Ann. Intern. Med..

[27] Kramer A.F., Larish J.F., Strayer D.L. (1995). Training for attentional control in dual task settings: A comparison of young and old adults. J. Exp. Psychol. Appl..

[28] Patelaki E., Foxe J.J., Mantel E.P., Kassis G., Freedman E.G. (2023). Paradoxical improvement of cognitive control in older adults under dual-task walking conditions is associated with more flexible reallocation of neural resources: A Mobile Brain-Body Imaging (MoBI) study. Neuroimage.

[29] Wu T., Spagna A., Mackie M.A., Fan J. (2023). Resource sharing in cognitive control: Behavioral evidence and neural substrates. Neuroimage.

[30] Lundin-Olsson L., Nyberg L., Gustafson Y. (1997). “Stops walking when talking” as a predictor of falls in elderly people. Lancet..

[31] Beauchet O., Annweiler C., Allali G., Berrut G., Dubost V. (2008). Dual task-related changes in gait performance in older adults: A new way of predicting recurrent falls?. J. Am. Geriatr. Soc..

[32] Pettersson A.F., Olsson E., Wahlund L.O. (2007). Effect of divided attention on gait in subjects with and without cognitive impairment. J. Geriatr. Psychiatry Neurol..

[33] Manckoundia P., Pfitzenmeyer P., d’Athis P., Dubost V., Mourey F. (2006). Impact of cognitive task on the posture of elderly subjects with Alzheimer’s disease compared to healthy elderly subjects. Mov. Disord..

[34] Beauchet O., Dubost V., Herrmann F., Rabilloud M., Gonthier R., Kressig R.W. (2005). Relationship between dual-task related gait changes and intrinsic risk factors for falls among transitional frail older adults. Aging Clin. Exp. Res..

[35] Hausdorff J.M., Yogev G., Springer S., Simon E.S., Giladi N. (2005). Walking is more like catching than tapping: Gait in the elderly as a complex cognitive task. Exp. Brain Res..

[36] Plummer-D’Amato P., Altmann L.J., Saracino D., Fox E., Behrman A.L., Marsiske M. (2008). Interactions between cognitive tasks and gait after stroke: A dual task study. Gait Posture.

[37] Marusic U., Taube W., Morrison S.A., Biasutti L., Grassi B., De Pauw K., Meeusen R., Pisot R., Ruffieux J. (2019). Aging effects on prefrontal cortex oxygenation in a posture-cognition dual-task: An fNIRS pilot study. Eur. Rev. Aging Phys. Act..

[38] Ohara K., Kuriyama C., Hada T., Suzuki S., Nakayama Y., Abo M. (2021). A pilot study verifying the effectiveness of high-frequency repetitive transcranial magnetic stimulation in combination with intensive speech-language-hearing therapy in patients with chronic aphasia. NeuroRehabilitation.

